# Effect of High Altitude on Small Pulmonary Vein and Artery Volume in the COPDGene Cohort: Towards Better Understanding of Lung Physiology and Pulmonary Disease

**DOI:** 10.3390/jpm15080377

**Published:** 2025-08-15

**Authors:** Anastasia K. A. L. Kwee, Esther Pompe, Leticia Gallardo Estrella, Jean-Paul Charbonnier, Stephen M. Humphries, Harm A. W. M. Tiddens, James D. Crapo, Richard Casaburi, Pim A. de Jong, David A. Lynch, Firdaus A. A. Mohamed Hoesein

**Affiliations:** 1 Department of Radiology, University Medical Center Utrecht and Utrecht University, 3584 CX Utrecht, The Netherlandsp.dejong-8@umcutrecht.nl (P.A.d.J.);; 2 Thirona B.V., 6525 EC Nijmegen, The Netherlandsjeanpaulcharbonnier@thirona.eu (J.-P.C.);; 3 Department of Radiology, National Jewish Health, Denver, CO 80206, USA; 4 Department of Pulmonology, National Jewish Health, Denver, CO 80206, USA; 5 Division of Respiratory and Critical Care Physiology and Medicine, The Lundquist Institute for Biomedical Innovation at Harbor-UCLA Medical Center, Torrance, CA 90502, USA; casaburi@ucla.edu

**Keywords:** pulmonary veins, chronic obstructive pulmonary disease, high altitude, computed tomography

## Abstract

**Background:** To personalize the care for persons with smoking-related lung disease, a thorough understanding of its etiology is essential. The role of pulmonary vessels remains poorly understood. Living at high altitude provides a natural model to investigate the effects of low oxygen levels on pulmonary vessels. This study aims to evaluate the relationship between living at high altitudes and small pulmonary vein and artery volumes. We hypothesize that small vein and artery volumes were independently associated with living at high altitude. **Methods:** We quantified small pulmonary vein and artery dimensions (ᴓ < 1 mm) on computed tomography (CT) down to 0.2 mm in diameter and normalized the dimensions by body surface area. In 8931 current and former smokers participating in the COPDGene study, we used multivariate regression models corrected for clinical and technical confounders. **Results:** 1262 residents (14.1%) were defined as high-altitude residents (~1600 m, Denver, CO, USA). Compared to lower-altitude residents, the high-altitude residents had a higher age (62.0 ± 9.1 vs. 59.6 ± 9.0 years), more pack-years smoked (46.8 vs. 44.1) and a lower FEV_1_% predicted (64.6 ± 32.4% vs. 76.8 ± 25.2%). Both mean small artery volume (4.09 ± 0.89 mL/m^2^ vs. 3.85 ± 0.90 mL/m^2^) and mean small vein volume (2.96 ± 0.53 mL/m^2^ vs. 2.67 ± 0.53 mL/m^2^) were higher in high-altitude residents. Multivariate linear regression showed that, in those without COPD, high-altitude residents have a higher small vein volume (0.129 mL/m^2^, *p* < 0.001) and higher small artery volume (0.170 mL/m^2^, *p* = 0.001) compared to lower-altitude residents. There was no significant association in residents with COPD. **Conclusions:** In current and former smokers without COPD, higher small pulmonary vein and artery volumes were associated with living at high altitude, independent of lung disease or technical CT parameters. A potential cause includes vascular remodeling due to an elevated need for blood oxygen transport, which becomes concealed when COPD develops.

## 1. Background

Residents of high-altitude regions live in an environment with a lower partial pressure of oxygen compared to regions at sea level. Consequently, these individuals developed physiological and anatomical changes to adapt to an environment of chronic low oxygen. These changes include polycythemia, hypervolemia and pulmonary hypertension [1,2]. For personalized medicine of lung disease in people who smoke, the high altitude provides a ‘natural experiment’ to investigate its effects on pulmonary veins and arteries. Although it has been hypothesized that vascular remodeling is important in the etiology of smoking related lung disease, its exact role remains poorly understood.

Peruvian research teams performed extensive research into the cardiovascular physiology of high-altitude natives [3]. Their cardiac catheterization studies reported evidence of mild pulmonary hypertension and increased pulmonary vascular resistance in healthy high-altitude natives [2]. Subsequent histological studies in the same population showed that these findings can be attributed to increased amounts of muscularity in the walls of the small pulmonary arteries and arterioles, induced by chronic exposure to hypoxia [4,5]. Similar findings were reported on the pulmonary veins in post mortem studies on native highlanders by Wagenvoort and colleagues. They observed medial thickening of the small pulmonary veins [6,7], which has also been described in animal studies on high-altitude cattle or rats in a hypoxic environment [8,9].

To date, no in vivo studies have been performed to assess the pulmonary vascular system in individuals residing at high altitude, mainly because it has been very challenging to assess small pulmonary vessels, and especially veins, in vivo. Our group has recently shown that it is possible to measure pulmonary vessels down to 0.2 mm in diameter on non-contrast chest computed tomography (CT), whilst also differentiating pulmonary veins from arteries [10]. In a subsequent study, we found associations between higher pulmonary vein volumes and lower oxygen saturation in smokers with and without COPD.

In this study, we investigate small pulmonary veins and arteries in vivo in high-altitude participants from the COPDGene cohort by comparing participants from Denver (CO, USA, ~1600 m) to participants who are living at lower altitudes (~0–400 m). We hypothesized that high-altitude residents have higher volumes of small pulmonary arteries and veins compared to lower-altitude residents.

## 2. Methods

### 2.1. Study Population

We included a subset of phase 1 participants of the COPDGene study (ClinicalTrials.gov: NCT00608764) [11]. All participants provided written informed consent. The full study protocol including inclusion and exclusion criteria is available at www.copdgene.org. The COPDGene study was approved by the institutional review boards at each of the 21 clinical sites (Table S1).

Subjects enrolled in Denver, Colorado (~1600 m/~5200 feet above sea level) were classified as the high-altitude group. Residents enrolled at other study centers (~0–400 m above sea level) were classified as the lower-altitude group.

### 2.2. Spirometry and Clinical Data

Spirometry data were collected using a standardized protocol and spirometer (ndd EasyOne™ Spirometer, Zurich, Switzerland). Details are described in a previous paper [11]. Post-bronchodilator forced expiratory volume in 1 s (FEV_1_) and forced vital capacity (FVC) are presented as a percentage of their predicted values (% predicted) [12] The pulmonary function test results were categorized according to the Global Initiative for Obstructive Lung Disease (GOLD) as stage 0–4 or Preserved Ratio Impaired Spirometry (PRISm) [13].

Race and cigarette smoking pack-years were self-reported. Height and weight were measured and body mass index (BMI) was calculated. Functional exercise performance was assessed using the 6-min walking distance (6MWD) test. Activity-related dyspnoea was self-assessed using the modified Medical Research Council (mMRC) dyspnoea scale [14].

Oxygen saturation was measured using a pulse oximeter on a finger without nail polish, after the participant had been at rest in seated position for >5 min. In participants with supplemental oxygen use, the oxygen was discontinued during the saturation measurement. If oxygen saturation fell below 82%, the supplemental oxygen was restarted and a reading of 82% was registered [15].

### 2.3. CT Acquisition and Measurements

CT scan protocols have been described previously [11]. Briefly, multidetector CT scanners (≥16 detector channels) were used to acquire thin slice non-contrast-enhanced volumetric CT scans at inspiration (200 mAs) and at the end of expiration (50 mAs). Coronary artery calcification was quantified using the Agatston algorithm on ungated CT scans, hereafter referred to as the ‘modified Agatston’ (mAgatston) [16]. Severe-to-extensive coronary artery disease was defined as >300 Agatston Units, similar to the CAD-RADS™ 2.0 classification [17]. Emphysema was expressed as the percentage of voxels below −950 Hounsfield Units (HU) on inspiratory CT scans (%LAA950). Air trapping was quantified on expiratory CT scans and expressed as the percentage of voxels below −856 HU. Airway wall thickness was expressed as the square root wall area of an airway with a diameter of 10 mm (Pi10) [18]. All quantitative CT analyses were performed using Thirona’s artificial intelligence (AI)-based lung quantification platform LungQ (Thirona, The Netherlands, http://www.thirona.eu, accessed on 20 May 2025). Emphysema was also visually assessed by expert readers according the Fleischner Society recommendations as none, trace, mild, moderate, confluent or advanced destructive [19].

### 2.4. CT Pulmonary Vein and Artery Quantification

The quantification method has been published previously [10]. In short, the artery–vein phenotyping analysis (AVX) was performed using the lung quantification platform LungQ^®^ AVX (Thirona, Nijmegen, The Netherlands). The software has three components: (1) voxel-wise segmentation of pulmonary arteries and veins, (2) separation of the identified vascular tree into individual branches, and (3) quantification of vascular diameters and volumes for each branch. Small vein volume was quantified as the total volume of all pulmonary venous branches with a diameter < 1 mm, and small artery volume as the total volume of all pulmonary arterial branches with a diameter < 1 mm. To accommodate for differences in body size, small vein volume and small artery volume were normalized by dividing it by body surface area, which was calculated using the DuBois method (BSA [m^2^] = 0.007184 × Height [cm]^0.725^ × Weight [kg]^0.425^) [20].

### 2.5. Statistical Analysis

Continuous variables were presented as mean ± standard deviation or median (IQR). Missing values were present in 12 variables. In nine variables, the percentage of missing values was <1%. In three variables, the percentages of missing data were 7.8%, 11.5% and 11.5%. Missing data were imputed with multiple imputation (function mice from package mice (version 3.16.0)). Data were imputed using 10 iterations and 10 imputed datasets were generated.

Multivariable linear regression was used to analyze the association between small vein or artery volume and high altitude, corrected for age, gender, race, BMI, FEV_1_% predicted, supplemental oxygen use, smoking status, pack-years smoked, mMRC dyspnea score, 6MWD, %LAA950, Pi10, mAgatston score, severe exacerbations, pixel spacing and scanner model. All analyses were performed with R version 4.3.3.

## 3. Results

### 3.1. Subject Characteristics

The study population consisted of 8931 current and former smokers classified according to the GOLD severity stages. In total, 4885 without COPD (i.e., GOLD 0 + PRISm) and 4046 with COPD were included. A total of 1262 (14.1%) residents were classified as the high-altitude group (~1600 m), the remaining 7669 (85.9%) residents were classified as the lower-altitude group (~0–400 m). Univariate comparison showed that the high-altitude group had a higher mean age (62.0 ± 9.1 vs. 59.6 ± 9.0 years), more pack-years smoked (46.8 ± 25.8 years vs. 44.1 ± 24.9 years) and a lower FEV_1_% predicted (64.6 ± 32.4% vs. 76.8 ± 25.2%) compared to the lower-altitude group (Table 1). High-altitude residents had a lower resting oxygen saturation compared to lower-altitude residents (94% [91–96%]) vs. 97% [96–98%]). Additionally, residents at high altitude showed a higher prevalence of more severe COPD, with 28.8% in GOLD stages 3 and 4, compared to 15.8% in the lower-altitude residents. Similarly, moderate and confluent emphysema was more common in high-altitude residents, affecting 38.2%, compared to 24.2% in the lower-altitude group.

### 3.2. Univariate and Multivariate Analysis

In univariate analysis for all subjects, both mean small vein volume (2.96 ± 0.53 mL/m^2^ vs. 2.67 ± 0.53 mL/m^2^) and mean small artery volume (4.09 ± 0.89 mL/m^2^ vs. 3.85 ± 0.90 mL/m^2^) were higher in high-altitude residents compared to lower altitude residents. For subjects with COPD (GOLD I-IV), high-altitude residents also had higher small vein volumes (3.03 ± 0.60 mL/m^2^ vs. 2.83 ± 0.57 mL/m^2^) and small artery volumes (4.06 ± 0.85 mL/m^2^ vs. 4.01 ± 0.92 mL/m^2^) vs. lower-altitude residents.

Multivariable linear regression showed that, in subjects without COPD, high-altitude residents have both a higher small pulmonary vein volume (0.129 mL/m^2^, *p* < 0.001) and higher small pulmonary artery volume (0.170 mL/m^2^, *p* = 0.001) compared to lower-altitude residents (Table 2 and Table 3). Restricting the analysis to individuals with COPD GOLD 1–4, the association was not present for either small pulmonary vein volume (0.031 mL/m^2^, *p* = 0.14) or small pulmonary artery volume (0.001 mL/m^2^, *p* = 0.98) (Table 4 and Table 5).

## 4. Discussion

In this study we found that high-altitude residents without COPD have a significantly higher small pulmonary vein and artery volume, after adjustment for covariates including smoking status, pulmonary function, emphysema and technical covariates. We did not observe this difference in high-altitude residents with COPD. Our findings add insight into the pathophysiology of small pulmonary vessels in relation to living at high altitude and chronic low oxygen inhalation and correspond favorably to previous histological findings in humans and in animal studies. It is important to consider altitude as a critical factor when interpreting these venous and arterial dimensions in future studies on the small pulmonary vessels. Additionally, these findings may influence personalized pulmonary medicine of high-altitude-related pulmonary vascular disease, by potentially targeting vascular remodeling and adjusting for the presence of COPD.

Our results show a higher small pulmonary vein volume in high-altitude residents without COPD. Only a few authors have studied the morphology of the pulmonary veins in relation to living at high altitude and chronic low oxygen inhalation (Table 6). In 1982, Wagenvoort et al. performed an autopsy study on healthy adults born and residing in the Andean mountains at altitudes of 3400–4400 m. Compared to sea-level residents, more than half of the high-altitude residents showed evident hypertrophy of the pulmonary venous medial layer [7]. These changes are sometimes referred to as arterialization of veins, as the changes in the wall resemble a muscular pulmonary artery. In line with these observations, animal studies of both cattle raised at high altitudes and of rats exposed to hypoxia found an increase in smooth muscle cells of the small pulmonary veins or hypertrophy of the venous medial layer [8,9]. Additionally, considering different organ systems, a study on the retinal vessels of mountaineers have found that dilation occurs in the retinal veins at 5850 m altitude [21]. This effect was even stronger during exercise after acclimatization, suggesting that an increase in blood flow is required in hypoxic conditions. These findings align with our observed increase in small pulmonary vein volumes in high-altitude residents, which could be explained by thickening due to venous remodeling and/or by an increased demand for blood oxygen transport, and therefore an increased blood volume. We observed a small, although statistically significantly higher small pulmonary vein volume in high-altitude residents after adjustment for many covariates. This small difference may be explained by the fact that the altitude in our study was much lower than in the studies discussed above.

Small pulmonary arteries respond to low oxygen levels in a manner similar to that of pulmonary veins: higher volumes in the high-altitude residents without COPD. Several studies of native highlanders described hypertrophy of the arterial medial layer, mainly due to an increased amount of smooth muscle cells and muscularization [3,4,5,7]. The same findings have also been reported in animal studies of high-altitude-raised cattle or rats in a hypoxic environment [8,9]. Similar to the veins, medial hypertrophy might be an explanation for the observed increase in small artery volume. Another possible explanation for the higher volumes in the small arteries is hypoxic pulmonary vasoconstriction (HPV). This mainly occurs in the pulmonary arteries in response to low oxygen levels in the alveoli, diverting the blood to areas of the lung with better ventilation to optimize gas exchange and ventilation/perfusion ratio [22,23,24].

However, when ventilation is low in the whole lung, like in high-altitude regions, the ability of HPV to improve ventilation/perfusion ratios is reduced [25]. In situations where ventilation is uniformly reduced, HPV instead allows for recruitment of previously under-perfused capillaries, increasing the surface area for gas exchange. This recruitment increases the total amount of capillaries involved in gas exchange and thus could explain increased vessel volume for both small arteries and veins [26].

Lastly, another possibility is that constriction of the smallest pulmonary arteries, below 0.2 mm in diameter, contributes to the observed arterial volume increase. However, since these smallest arteries were not measured in our study, their role in the volume increase remains speculative.

It is remarkable that, in our multivariable models, we observed a significant increase in venous and arterial volumes in high-altitude residents without COPD, but not in those with COPD. Apparently, COPD affects both the small pulmonary veins and arteries. Our finding might be explained by several physiological differences between non-COPD and COPD subjects. First, chronic inflammation in COPD causes vascular remodeling, including deposition of extracellular matrix in the intima, which decreases vascular compliance and limits capacity for volume expansion [27,28,29]. Second, in COPD, hypoxic pulmonary condition often leads to elevated pulmonary pressures that further restrict vascular dilation, whereas non-COPD individuals show a more balanced response without this restriction [30]. Third, loss of pulmonary vessels in COPD has been described previously and could explain why there is no visible volume increase in high-altitude residents with COPD [27]. Additionally, physiological and demographic factors can influence pulmonary vascular structure and COPD risk. Gender and race differences may reflect underlying anatomical or genetic variability, while BMI may impact vascular volume through effects on body composition and hemodynamics. These variables were included as covariates in our models to account for their potential confounding effects. These factors combined could explain why the small venous and arterial volumes become similar between high-altitude residents and lower-altitude residents with COPD, although further investigation is needed to understand the vascular component of COPD.

While the pulmonary vasculature is the main focus of this study, our data also show that high-altitude subjects enrolled in COPDGene had a lower pulmonary function and a higher prevalence of moderate-to-severe emphysema compared to lower-altitude residents. This could be due to selective recruitment of subjects with more severe COPD at this center. Previous studies on COPD subjects at high altitudes reported a decreased exercise capacity and more frequent supplemental oxygen use, but whether there is a direct relationship between COPD prevalence and residing at high altitudes remains inconclusive [31,32].

Our findings suggest that CT-quantified small pulmonary artery and vein volumes could be used as early biomarkers of vascular remodeling, particularly in patients exposed to chronic hypoxia. Chronic exposure to hypoxia at high altitude may lead to structural and functional changes in the pulmonary vasculature, including remodeling that can influence pulmonary pressures and gas exchange efficiency. By characterizing these adaptations—particularly in individuals without overt cardiopulmonary disease—we may identify early vascular changes that could be modifiable. This insight could support the development of therapies aimed at preventing or mitigating maladaptive remodeling, especially in at-risk populations such as smokers or those with borderline pulmonary function. Moreover, therapeutic approaches informed by high-altitude physiology could potentially be translated to other hypoxia-driven conditions, including chronic obstructive pulmonary disease (COPD) and pulmonary hypertension.

This study has several limitations. First, our study included residents residing at an altitude of approximately 1600 m, which is lower than the 4000–5000 m seen in most previous studies on high-altitude populations. As a result, the effect that we observed at this altitude may not be as strong as it would be at higher elevations. Second, we do not have data on the duration of residency in the high-altitude residents, nor do we have precise altitudes for the actual residences of the participants. Physiological alterations at high altitudes may vary between short-term exposure compared to long-term residency and therefore the effect we measured might be attenuated [33].

Third, we have no histological proof for our findings. Therefore, it is not possible to assess whether the observed increase in venous and arterial volume is due to changes in the vessel wall, lumen or surrounding tissue. Heart failure could be another explanation for our findings, as it can lead to increased blood volume in the small veins. Likely of relevance, histological studies have shown an increase in vessel wall thickness in high-altitude residents [7].

Fourth, our CT-based analysis is not able to distinguish functional from structural changes, making it unclear whether the observed vascular changes reflect transient hypoxia-induced vasodilation or chronic structural remodeling.

Lastly, while mechanisms such as vascular remodeling and hypoxic pulmonary vasoconstriction may underlie the observed associations, our study did not include functional or biochemical data to directly assess these pathways. These interpretations are therefore hypothesis-generating and highlight the need for future research incorporating physiological and molecular measurements to elucidate the mechanisms involved.

In conclusion, we found that individuals with a smoking history but normal spirometry residing at high altitudes exhibited increased small pulmonary vein and artery volumes compared to low-altitude residents. Potential underlying mechanisms include vascular structural remodeling and/or an increased requirement for oxygen transport and blood flow. The study findings could be relevant for personalized pulmonary medicine, as the contribution of pulmonary small vessels is currently underappreciated.

## Figures and Tables

**Table 1:** Clinical characteristics of the study population.

	High Altitude Residents (N = 1262)	Lower Altitude Residents (N = 7669)
*Age (yr)*	62.0 ± 9.1	59.6 ± 9.0
*Male gender (N, %)*	650 (51.5%)	4060 (52.9%)
*BMI (kg/m^2^)*	27.9 ± 6.1	28.9 ± 6.3
*Packyears (yr)*	46.8 ± 25.8	44.1 ± 24.9
*Non-Hispanic White residents (N, %)*	1095 (86.8%)	5063 (66.0%)
*Black or African-American residents (N, %)*	167 (13.2%)	2606 (34.0%)
*Current smokers (n,%)*	447 (35.4%)	4080 (53.2%)
*Spirometry*		
*FEV_1_ (% predicted)*	64.6 ± 32.4	76.8 ± 25.2
*FVC (% predicted)*	86.7 ± 20.0	87.0 ± 17.9
*6MWD (m)*	433 ± 135	409 ± 120
*Resting SpO_2_ (%)*	94 (91–96)	97 (96–98)
*mMRC dyspnea scale*	1 (0–3)	1 (0–3)
*Supplemental oxygen users (N, %)*	463 (36.7%)	577 (7.5%)
*CT quantified parameters*		
*Emphysema (%)*	3.2 (0.8–15.2)	2.0 (0.5–6.7)
*Air trapping (%)*	16.1 (5.0–43.8)	14.2 (6.39–29.9)
*Pi10 (mm)*	2.35 ± 0.63	2.35 ± 0.62
*mAgatston score*	11 (0–164)	14 (0–179)
*CT Vascular parameters*		
*Mean small vein volume (mL/m^2^)*	2.96 ± 0.53	2.67 ± 0.53
*Mean small artery volume (mL/m^2^)*	4.09 ± 0.89	3.85 ± 0.90
*Min-max (IQR) of small vein volume (mL/m^2^)*	0.28–5.29 (2.52, 3.33)	0.73–6.07 (2.30, 2.99)
*Min-max (IQR) of small artery volume (mL/m^2^)*	2.01–7.68 (3.45, 4.61)	1.58–8.18 (3.20, 4.40)
*Emphysema visual score (N, %)*		
*0—none*	296 (23.5%)	2647 (34.5%)
*1—* *trace*	167 (13.2%)	1426 (18.6%)
*2—* *mild*	239 (18.9%)	1511 (19.7%)
*3—* *moderate*	248 (19.7%)	1183 (15.4%)
*4—* *confluent*	233 (18.5%)	672 (8.8%)
*5—* *advanced destructive*	79 (6.3%)	230 (3.0%)
*COPD GOLD* *stage (N, %)*		
*PRISm*	94 (7.4%)	980 (12.8%)
*0*	420 (33.3%)	3390 (44.2%)
*1*	109 (8.6%)	597 (7.8%)
*2*	275 (21.8%)	1485 (19.4%)
*3*	230 (18.2%)	824 (10.7%)
*4*	134 (10.6%)	393 (5.1%)

Legend: Data given are mean ± standard deviation, median and interquartile range in parentheses, or number and percentage in parenthesis, depending on the data distribution. BMI is body mass index, FEV_1_ is forced expiratory volume in one second, FVC is forced vital capacity, SpO_2_ is peripheral oxygen saturation, CT is computed tomography, Pi10 is square root of wall area for a theoretical airway with a lumen of 10 mm, 6MWD is six-minute walking distance, mMRC is modified Medical Research Council dyspnea scale, COPD is chronic obstructive pulmonary disease, GOLD is global initiative for chronic obstructive lung disease, PRISm is preserved ratio impaired spirometry.

**Table 2:** Multivariable linear regression for analysis of the association between living at high altitudes and small vein volume in subjects without COPD.

Determinant	Unit Change	B	95% CI	T Statistic	*p* Value
High altitude	Yes/no	0.129	[0.075, 0.182]	4.69	<0.001
Supplemental oxygen use	Yes/no	0.054	[−0.029, 0.138]	1.28	0.20
Age	Per year	−0.001	[−0.002, 0.011]	−0.71	0.50
BMI	Per kg/m^2^	−0.018	[−0.020, −0.017]	−19.0	<0.001
Gender	For females	−0.056	[−0.083, −0.029]	−4.11	<0.001
Race	For African Americans	−0.276	[−0.303, −0.249]	−19.8	<0.001
Pack-years smoked	Per pack-year	0.001	[0.001, 0.002]	4.08	<0.001
Smoking status	For current smokers	0.107	[0.079, 0.134]	7.63	<0.001
FEV_1_ % predicted	Per % predicted	0.002	[0.002, 0.003]	7.79	<0.001
Emphysema	Per % predicted	0.015	[−0.007, 0.037]	1.34	0.18
Pi10	Per mm	−0.011	[−0.036, 0.014]	−0.86	0.39
Coronary calcium	Per mAgatston unit	0.017	[0.005, 0.029]	2.84	0.005
6MWD	Per 100 m	0.005	[−0.006, 0.017]	0.89	0.37
Severe exacerbations	For severe exacerbations	0.035	[−0.014, 0.084]	1.40	0.16
mMRC Dyspnea Score 1	For score 1	0.005	[−0.027, 0.036]	0.27	0.78
mMRC Dyspnea Score 2	For score 2	−0.007	[−0.045, 0.030]	−0.39	0.70
mMRC Dyspnea Score 3	For score 3	0.035	[−0.001, 0.071]	1.90	0.06
mMRC Dyspnea Score 4	For score 4	0.007	[−0.047, 0.061]	0.26	0.79

Legend: BMI is body mass index, FEV_1_ is forced expiratory volume in one second, Pi10 is square root of wall area for a theoretical airway with a lumen of 10 mm, 6MWD is six-minute walking distance, mMRC is modified Medical Research Council dyspnea scale. Pixel spacing, CT scanner models and research center were also added as covariates.

**Table 3:** Multivariable linear regression for analysis of the association between living at high altitudes and small vein volume in subjects with COPD.

Determinant	Unit Change	B	95% CI	T Statistic	*p* Value
High altitude	Yes/no	0.031	[−0.011, 0.072]	1.47	0.14
Supplemental oxygen use	Yes/no	0.125	[0.091, 0.159]	7.12	<0.001
Age	Per year	−0.001	[−0.003, 0.000]	−1.77	0.08
BMI	Per kg/m^2^	−0.023	[−0.025, −0.022]	−28.4	<0.001
Gender	For females	−0.084	[−0.106, −0.063]	−7.65	<0.001
Race	For African Americans	−0.261	[−0.283, −0.237]	−22.2	<0.001
Pack-years smoked	Per pack-year	0.002	[0.001, 0.002]	8.07	<0.001
Smoking status	For current smokers	0.098	[0.076, 0.120]	8.73	<0.001
FEV_1_ % predicted	Per % predicted	<−0.001	[−0.001, 0.001]	1.29	0.20
Emphysema	Per % predicted	0.109	[0.093, 0.125]	13.1	<0.001
Pi10	Per mm	0.021	[0.003, 0.039]	2.26	0.02
Coronary calcium	Per mAgatston unit	0.017	[0.008, 0.026]	3.59	<0.001
6MWD	Per 100 m	−0.005	[−0.014, 0.005]	−1.00	0.32
Severe exacerbations	For severe exacerbations	0.004	[−0.026, 0.033]	0.23	0.81
mMRC Dyspnea Score 1	For score 1	0.012	[−0.015, 0.039]	0.88	0.38
mMRC Dyspnea Score 2	For score 2	0.015	[−0.014, 0.045]	1.01	0.31
mMRC Dyspnea Score 3	For score 3	0.055	[0.027, 0.083]	3.83	<0.001
mMRC Dyspnea Score 4	For score 4	0.055	[0.019, 0.092]	2.96	0.003

Legend: BMI is body mass index, FEV_1_ is forced expiratory volume in one second, Pi10 is square root of wall area for a theoretical airway with a lumen of 10 mm, 6MWD is six-minute walking distance, mMRC is modified Medical Research Council dyspnea scale. Pixel spacing, CT scanner models and research center were also added as covariates.

**Table 4:** Multivariable linear regression for analysis of the association between living at high altitudes and small artery volume in subjects without COPD.

Determinant	Unit Change	B	95% CI	T Statistic	*p* Value
High altitude	Yes/no	0.170	[0.068, 0.272]	3.26	0.001
Supplemental oxygen use	Yes/no	−0.016	[−0.174, 0.143]	−0.19	0.85
Age	Per year	−0.005	[−0.008, −0.002]	−3.10	0.002
BMI	Per kg/m^2^	−0.013	[−0.017, −0.009]	−7.10	<0.001
Gender	For females	−0.113	[−0.164, −0.062]	−4.34	<0.001
Race	For African Americans	−0.672	[−0.724, −0.620]	−25.5	<0.001
Pack-years smoked	Per pack-year	0.004	[0.003, 0.005]	7.21	<0.001
Smoking status	For current smokers	0.285	[0.233, 0.337]	10.7	<0.001
FEV_1_ % predicted	Per % predicted	0.003	[0.002, 0.004]	4.58	<0.001
Emphysema	Per % predicted	0.022	[−0.020, 0.063]	1.01	0.31
Pi10	Per mm	−0.087	[−0.134, −0.040]	−3.61	<0.001
Coronary calcium	Per mAgatston unit	0.037	[0.015, 0.060]	3.24	0.001
6MWD	Per 100 m	0.027	[0.005, 0.049]	2.38	0.02
Severe exacerbations	For severe exacerbations	0.068	[−0.026, 0.161]	1.42	0.16
mMRC Dyspnea Score 1	For score 1	−0.005	[−0.066, 0.056]	−0.16	0.87
mMRC Dyspnea Score 2	For score 2	0.001	[−0.070, 0.072]	0.02	0.98
mMRC Dyspnea Score 3	For score 3	0.016	[−0.052, 0.084]	0.45	0.65
mMRC Dyspnea Score 4	For score 4	0.008	[−0.093, 0.110]	0.16	0.88

Legend: BMI is body mass index, FEV_1_ is forced expiratory volume in one second, Pi10 is square root of wall area for a theoretical airway with a lumen of 10 mm, 6MWD is six-minute walking distance, mMRC is modified Medical Research Council dyspnea scale. Pixel spacing, CT scanner models and research center were also added as covariates.

**Table 5:** Multivariable linear regression for analysis of the association between living at high altitudes and small artery volume in subjects with COPD.

Determinant	Unit Change	B	95% CI	T Statistic	*p* Value
High altitude	Yes/no	0.001	[−0.071, 0.073]	0.03	0.98
Supplemental oxygen use	Yes/no	0.034	[−0.026, 0.095]	1.12	0.26
Age	Per year	−0.005	[−0.007 −0.003]	−4.11	<0.001
BMI	Per kg/m^2^	−0.016	[−0.019, −0.013]	−11.2	<0.001
Gender	For females	−0.139	[−0.177, −0.101]	−7.22	<0.001
Race	For African Americans	−0.655	[−0.695, −0.615]	−31.9	<0.001
Pack-years smoked	Per pack-year	0.003	[0.002, 0.004]	8.81	<0.001
Smoking status	For current smokers	0.350	[0.311, 0.389]	17.8	<0.001
FEV_1_ % predicted	Per % predicted	0.002	[0.001, 0.003]	4.17	<0.001
Emphysema	Per % predicted	0.096	[0.068, 0.125]	6.65	<0.001
Pi10	Per mm	−0.022	[−0.055, 0.010]	−1.37	0.17
Coronary calcium	Per mAgatston unit	0.035	[0.019, 0.051]	4.24	<0.001
6MWD	Per 100 m	0.015	[−0.014, 0.032]	1.79	0.07
Severe exacerbations	For severe exacerbations	0.049	[−0.003, 0.101]	1.84	0.07
mMRC Dyspnea Score 1	For score 1	0.005	[−0.043, 0.052]	0.20	0.84
mMRC Dyspnea Score 2	For score 2	−0.018	[−0.069, 0.034]	−0.76	0.50
mMRC Dyspnea Score 3	For score 3	0.012	[−0.037, 0.062]	0.49	0.63
mMRC Dyspnea Score 4	For score 4	0.052	[−0.013, 0.115]	1.57	0.12

Legend: BMI is body mass index, FEV_1_ is forced expiratory volume in one second, Pi10 is square root of wall area for a theoretical airway with a lumen of 10 mm, 6MWD is six-minute walking distance, mMRC is modified Medical Research Council dyspnea scale. Pixel spacing, CT scanner models and research center were also added as covariates.

**Table 6:** Overview of the literature on the effect of high-altitude residence or hypoxia on small pulmonary vascular volumes.

		Veins	Arteries
**Arias-Stella, 1963** Peruvian residents and sea-level residents	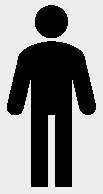	-	Distal arteries: more muscularization of medial layer Proximal arteries: hypertrophic medial layer
**Wagenvoort, 1982** 12 sea level adults, 12 high altitude adults (<3000 m)	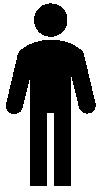	Venous medial hypertrophy	Arterial medial hypertrophy, especially in small arteries. Muscularization of non-muscular arterioles.
**Penaloza, 2007 (review)** Healthy highlanders	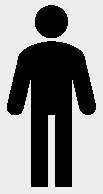	-	Thickening of the walls of arterioles in postnatal period, which remained in some through adulthood.
**Grover, 1963** Steers raised at high altitude (1000 m -> 1500 m -> 3800 m)	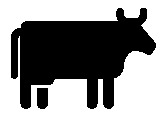	-	Rapidly increasing pulmonary arterial pressures at around 4000 m.
**Naeye, 1965** Dogs and cattle in hypoxic environment, infants with diaphragmatic defects	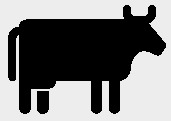	Hyperplasia and hypertrophy of medial SMC in small pulmonary veins	Dogs: hyperplasia of medial smooth muscle fibers Infants: more arterial muscle mass due to more cytoplasm in medial smooth muscle fibers.
**Jaenke, 1973** Cattle raised at high altitude	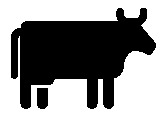	-	Arterial medial thickening
**Hunter, 1974** Rats in hypoxic environment	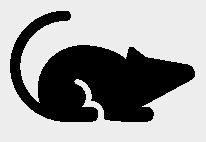	Vessels: no change in number of vessels, thickened (two elastic laminae with muscular coat in between)	
**Hislop, 1976** Rats in hypoxic environment	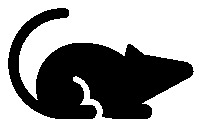	-	Decreased number of arteries with a diameter < 200 µm, increased arterial wall thickness
**Dingemans, 1978** Rats in hypoxic environment	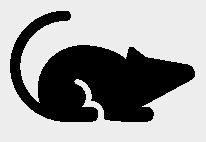	Veins and venules: medial hypertrophy (more layers of smooth muscle cells)	Medial hypertrophy of arteries and arterioles

Legend: overview of the literature on small pulmonary arteries and veins in high-altitude-related chronic hypoxia, or hypoxic environments.

## Data Availability

The datasets used and analyzed during the current study are available from the corresponding author on reasonable request. All shared data will be de-identified with respect to specific research subjects.

## Supplementary Materials

The following supporting information can be downloaded at: https://www.mdpi.com/article/10.3390/jpm15080377/s1, Table S1: COPDGene IRBs and Protocol Numbers.

## Abbreviations

6MWD	6-minute walking distance
AVX	artery–vein phenotyping analysis
BMI	body mass index
BSA	body surface area
COPD	chronic obstructive pulmonary disease
CT	computed tomography
FEV_1_% predicted	forced expiratory volume in 1 second as percent predicted
FVC% predicted	forced vital capacity as percent predicted
GOLD	global initiative for chronic obstructive lung disease
%LAA-950	percentage of low attenuation area below 950 Hounsfield Units
mMRC scale	modified Medical Research Council dyspnea scale
Pi10	square root of wall area for a theoretical airway with a lumen of 10 mm
PRISm	preserved ratio impaired spirometry
QCT	quantitative computed tomography
SpO_2_	oxygen saturation in arterial blood estimated by pulse oximetry
