# Supplementary material for: Effect of High Altitude on Small Pulmonary Vein and Artery Volume in the COPDGene Cohort: Towards Better Understanding of Lung Physiology and Pulmonary Disease
Source: J Pers Med. 2025 Aug 15;15(8):377. doi: 10.3390/jpm15080377 (PMC12387232; doi:10.3390/jpm15080377)
Supplement: Supplementary file 1 [file jpm-15-00377-s001.zip › jpm-3698074-supplementary.pdf]

Table S1. COPDGene IRBs and Protocol Numbers.

Clinical Center	Institution Title	Protocol Number
National Jewish Health	National Jewish IRB	HS-1883a
Brigham and Women's Hospital	Partners Human Research Committee	2007-P-000554/2; BWH
Baylor College of Medicine	Institutional Review Board for Baylor College of Medicine and Affiliated Hospitals	H-22209
Michael E. DeBakey VAMC	Institutional Review Board for Baylor College of Medicine and Affiliated Hospitals	H-22202
Columbia University Medical Center	Columbia University Medical Center IRB	IRB-AAAC9324
Duke University Medical Center	The Duke University Health System Institutional Review Board for Clinical Investigations (DUHS IRB)	Pro00004464
Johns Hopkins University	Johns Hopkins Medicine Institutional Review Boards (JHM IRB)	NA_00011524
Los Angeles Biomedical Research Institute	The John F. Wolf, MD Human Subjects Committee of Harbor-UCLA Medical Center	12756-01
Morehouse School of Medicine	Morehouse School of Medicine Institutional Review Board	07-1029
Temple University	Temple University Office for Human Subjects Protections Institutional Review Board	11369
University of Alabama at Birmingham	The University of Alabama at Birmingham Institutional Review Board for Human Use	FO70712014
University of California, San Diego	University of California, San Diego Human Research Protections Program	070876
University of Iowa	The University of Iowa Human Subjects Office	200710717
Ann Arbor VA	VA Ann Arbor Healthcare System IRB	PCC 2008-110732
University of Minnesota	University of Minnesota Research Subjects' Protection Programs (RSPP)	0801M24949
University of Pittsburgh	University of Pittsburgh Institutional Review Board	PRO07120059
University of Texas Health Sciences Center at San Antonio	UT Health Science Center San Antonio Institutional Review Board	HSC20070644H
Health Partners Research Foundation	Health Partners Research Foundation Institutional Review Board	07-127
University of Michigan	Medical School Institutional Review Board (IRBMED)	HUM00014973
Minneapolis VA Medical Center	Minneapolis VAMC IRB	4128-A
Fallon Clinic	Institutional Review Board/Research Review Committee Saint Vincent Hospital – Fallon Clinic – Fallon Community Health Plan	1143
